# Study protocol for 5Ways@School – An implementation and effectiveness trial of a school-based wellbeing intervention in 16 schools in Norway

**DOI:** 10.1177/14034948251370109

**Published:** 2025-09-08

**Authors:** Kristian Green Krogshus, Espen Bjertness, Nikolai Olavi Czajkowski, Rubén Rodriguez-Cano, Ragnhild Bang Nes

**Affiliations:** 1 Moss Municipality, Norway; 2 Promenta Research Centre, Department of Psychology, University of Oslo, Oslo, Norway; 3 Institute of Health and Society, Faculty of Medicine, University of Oslo, Oslo, Norway; 4 Norwegian Institute of Public Health, Oslo, Norway; 5 Department of Psychology, Norwegian University of Science and Technology, Trondheim, Norway; 6 Department of Philosophy, Classics, History of Art and Ideas, University of Oslo, Oslo, Norway

**Keywords:** Adolescent, child, mental health, loneliness, feasibility studies, health promotion

## Abstract

**Aims:**

This study aims to assess the effectiveness and implementation of the 5Ways@School curriculum-based intervention in Norwegian schools. The intervention builds on the Five Ways to Wellbeing framework, and promotes five action domains: connect with others, be physically active, take notice, keep learning, and give. The study objectives include assessing the intervention’s acceptability, appropriateness, feasibility, fidelity, and cost, as well as its impact on students’ wellbeing and mental health.

**Methods:**

The study is a hybrid effectiveness–implementation trial using mixed methods. It is a longitudinal, two-armed, non-randomized, controlled study involving students in grades 5 to 10 (ages 10 to 16). The primary outcomes are the intervention’s acceptability and feasibility, and its effectiveness on students’ wellbeing. Data collection includes web-based questionnaires for students and teachers, and qualitative interviews with teachers. Quantitative data will be analysed using multilevel regression models and network intervention analysis, while qualitative data will be analysed using thematic analysis.

**Conclusions:**

**The study aims to provide insights into the implementation and effectiveness of the wellbeing-promoting 5Ways@School intervention. The findings could inform the optimization of the 5Ways@School and other future school-based interventions, and thereby contribute to the wellbeing of children and young people. The study’s strengths include its real-life school setting and mixed methods approach, while limitations include its non-randomized design.**

**Trial registration:**

ClinicalTrials.gov, ID NCT06144502.

## Background

Mental health problems among children and adolescents are widespread and increasing globally [1–3]. This global challenge calls for accessible and effective treatment for existing mental disorders, preventive measures to avert the onset of mental health problems, and promotion of mental health and wellbeing for all children and young people. Schools are ideal settings for mental health promotion, since nearly all children can be reached there. School-based interventions can effectively improve mental health [4, 5], and the World Health Organization recommends teaching ‘life skills’ in schools – skills linked to mental wellbeing such as communication and self-awareness [6, 7]. Norway introduced the interdisciplinary topic ‘Health and Life Skills’ in 2020, covering mental and physical health, relationships, lifestyle, and more. This led to a need for evidence-based teaching materials, prompting the development of a new school intervention based on the Five Ways to Wellbeing framework.

### From the Five Ways to Wellbeing to 5Ways@School

In 2008, British scientists identified five key actions for wellbeing and mental health: connect with others, be physically active, take notice, keep learning, and give [8]. These ‘Five Ways to Wellbeing’ were grounded in positive psychology, particularly the broaden-and-build theory [9]. This theory suggests that positive emotions expand cognitive and behavioural capacities, thereby fostering long-term wellbeing. Extensive research supports the Five Ways [8], and practising them is associated with increased wellbeing in a dose-response pattern [10]. In a recent large-scale randomized controlled trial (RCT), the Five Ways showed promising effects in promoting wellbeing and mental health among Norwegian adults [11]. However, to the best of our knowledge, application of the Five Ways as an educational programme for students within a school setting has not yet been scientifically explored.

### Combining effectiveness and implementation

Evaluating the effectiveness of public health interventions is essential to advance health outcomes and optimize resource allocation. However, the effectiveness of an intervention depends heavily on its implementation. Pilot and feasibility studies are therefore carried out to refine an intervention, resolve uncertainties regarding its feasibility, assess initial effects, and/or explore plausible causal mechanisms of change [12]. Combining design components from effectiveness trials and implementation trials into hybrid effectiveness–implementation studies can lead to faster translational gains, better implementation strategies, and more useful information for decision makers [13]. Finally, such studies can pinpoint aspects of interventions and/or their implementation that require improvement.

## The objectives of this study

Our objectives are to:

1. Investigate the implementation of the 5Ways@School intervention in terms of acceptability, appropriateness, feasibility, fidelity, and implementation cost;
2. Study the students’ wellbeing and mental health from enrolment through the follow-up period, and to examine the intervention’s effectiveness on these outcomes;
3. Identify which students, based on socio-economic position, gender, school grade, and pre-intervention level of mental health symptoms, derive the most or least benefit from the intervention;
4. Explore mechanisms that may explain post-intervention improvements in students’ wellbeing; and
5. Investigate how the intervention can be improved and optimized.

## Methods

### Study design and data collection

Our study is a hybrid effectiveness–implementation trial, using mixed methods, incorporating both quantitative data from web-based questionnaires completed by students and teachers and qualitative data gathered from teacher interviews and questionnaires. It is a longitudinal, two-armed, controlled study, in which we administered an intervention to approximately half of the study population first. The remaining part of the study population functioned as a control group during the first seven weeks, before they received the intervention themselves.

The study is non-randomized, since power analyses indicated that a cluster-randomized controlled trial would require a far greater number of schools than were available. To compensate for the weakness of non-randomization, we opted for the quasi-experimental extended selection cohorts (ESC) design using a 12-month follow-up period. The ESC design is a cohort-longitudinal approach involving adjacent cohorts (in this case, school classes) measured repeatedly, previously used to evaluate the effects of school-based interventions [14, 15]. With the ESC approach, we can investigate intervention effects by comparing unexposed students (using data from November 2023) with exposed students (using data from November 2024).

The data collection took place at school, where students completed web-based questionnaires five times over a 12-month period from November 2023 to November 2024. A web-based questionnaire was distributed to the participating teachers immediately after they had finished the intervention period in the first quartile of 2024. Furthermore, the teachers were invited to participate in qualitative interviews in November and December 2024.

Questionnaire data were collected using «Nettskjema», a digital survey system developed by the University Centre for Information Technology (USIT) at the University of Oslo. These data were de-identified, stored, and analysed in USIT’s secure platform TSD, as described in the Supplementary File. Interviews were recorded on the interviewers’ mobile phones using a designated app and then uploaded to TSD.

### Setting, participants, and recruitment

Norwegian compulsory education includes primary school (grades 1–7, ages 6–12) and lower secondary school (grades 8–10, ages 13–16). After consulting with the Head of Schools in Moss Municipality regarding the grade levels most likely to benefit from the intervention, we decided to focus on students in grades 5 through 10, encompassing ages 10 to 16.

The pilot phase took place in Råde, a neighbouring municipality of Moss, where seven teachers tested the intervention in their school classes. These teachers then provided feedback directly to the publishing house. The teachers were generally positive and satisfied with the teaching material, but reported some difficulties with the digital platform, which they found confusing. They also recommended greater flexibility in customizing exercises and a preference for more active and engaging exercises. In response to their feedback, improvements were made in the digital platform and a teacher guide was produced. However, the publishing house did not adjust the exercises before the full-scale trial. Two other school classes in Råde completed the student questionnaire, which was later adjusted based on feedback from these students.

**Figure 1.**
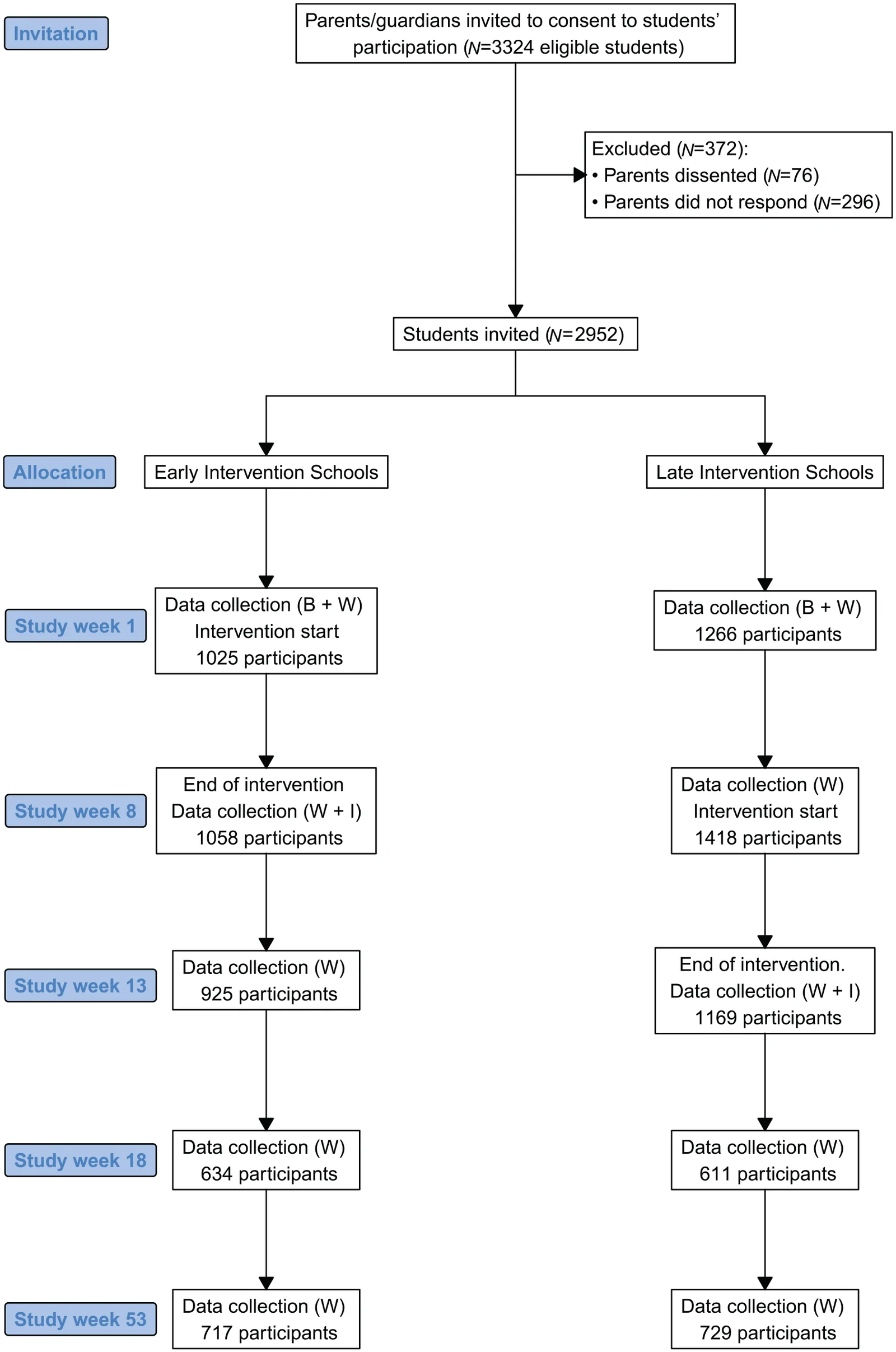
Flow chart of participating students, intervention periods, and data collections. Types of variables collected are indicated in parentheses: B=Background, I=Implementation, W=Wellbeing. Participants whose intervention allocation (early/late) is missing are not shown.

The full-scale trial was then run across all public schools in Moss, except for one school for 18 students with special needs. The teaching programme was integrated into regular school hours at the participating schools (*N*=16). The Head of Education in Moss Municipality mandated the inclusion of the teaching programme within the standard curriculum for all students in 5^th^ to 10^th^ grade. Participation in the data collection was voluntary and dependent on consent by students and their parents/guardians. All eligible students were invited to participate in the study (*N*=3324). The students were recruited through the schools’ digital information channel and invitations were sent to all parents. Of the total number of students eligible for participation (*N*=3324), 89% of the parents/guardians consented (*N*=2952), 2% dissented (*N*=76), and 9% did not respond to the invitation (*N*=296). The teachers then asked students whose parents had consented whether the students themselves would like to participate in the data collection. In total, 2437 students consented to participate in the first data collection, corresponding to 73% of the eligible population. During the 12-month study period, students were invited to complete five questionnaires. The median time to complete each student questionnaire was 10.4 minutes, while the median time for the teacher questionnaire was 5.0 minutes. Figure 1 provides a flow chart of participating students, intervention periods, and data collections.

### Intervention

The 5Ways@School intervention was a classroom-delivered teaching programme comprising two weekly school lessons for six consecutive weeks (12 school hours). The intervention was created in collaboration with Skolerom Publishing House, a Norwegian company producing teaching materials for primary and secondary education, and two of the authors of this paper (KGK and RBN). Since user involvement is crucial to ensure that an intervention is relevant and tailored to the specific needs of the school community [16], we invited teachers and students to participate in developing the intervention. Four teachers, four students, and personnel from the municipal school administration participated in workshops that we held. We also discussed our intervention and research project in meetings with the Norwegian Council for Mental Health, the Youth Participation Council in Moss Municipality, and the local branch of the association Mental Health. To ensure consistency and comparability across all school classes, teachers were instructed not to tailor the intervention to their specific context. This approach was intended to maintain the integrity of the intervention and allow for a clear assessment of its overall effectiveness. The intervention is described in the TIDieR checklist (see Supplementary Material).

### Ethics approval and consent to participate

Ethical approval was obtained from the Regional Committee for Medical and Health Research Ethics in South East Norway (reference number 491440). We initially planned to apply opt-out consent in our project to ensure a high response rate, but the Committee required active consent from parents. Thus, the municipal school administration sought informed consent from the parents before students were invited to complete questionnaires. Consent from one parent was considered adequate. If a student had one consenting and one dissenting parent, the dissenting parent was respected, and the student could not participate. Given consent from their parent(s), the students were invited and asked for informed consent. No remuneration or incentive was offered to students or their parents.

The teachers (*N*=156) were invited to complete a questionnaire (see Table I). Participation was voluntary and based on informed consent, and 113 teachers participated (72%). To ensure a decent response rate, we offered participating teachers the opportunity to win one of 10 gift vouchers of 1000 NOK (approximately 90 €).

**Table I.** Overview of measures of implementation.

Implementation outcome [18]	Student questionnaire items*	Teacher questionnaire items*
Acceptability (satisfaction with various aspects of the intervention)	I enjoyed learning about 5Ways@School. The teaching was easy to understand. The teaching was boring. The teaching was interesting. There were too few activities. There were too many activities. The teaching made me sad, afraid, or angry. The teaching made me happy.	I was well motivated to teach 5Ways@School. I enjoyed teaching 5Ways@School. Teaching life skills through 5Ways@School is a natural part of my job as a teacher. I got sufficient information and/or training before I started teaching 5Ways@School. There were too few activities. There were too many activities. Which of the five elements in 5Ways@School was easiest for you to teach? Which of the five elements in 5Ways@School was most challenging for you to teach?
Appropriateness (perceived fit, relevance, compatibility, and usefulness)	I learned something new that I can make use of. I will practise more of the Five Ways in the future. I think all students should learn about the Five Ways.	I think the students learned something new and useful. Which of the five elements in 5Ways@School do you think was most useful for the students? I think that 5Ways@School should be part of the ordinary school curriculum.
Fidelity (whether the intervention is delivered as intended; adherence to the intervention protocol)		I followed the teaching manual to a high degree. I completed all the six lessons in 5Ways@School. I had to abbreviate one or more lesson(s).

* The Likert scale format used for the statements was totally agree, partly agree, partly disagree, and totally disagree.

The teachers who taught the 5Ways@School programme were also invited to participate in qualitative interviews, and 20 teachers accepted the invitation. To ensure the teachers felt comfortable voicing their opinions, the interviews were conducted by two master-level psychology students unfamiliar to the teachers. This helped create a neutral environment, encouraging open and honest communication, thereby contributing to the validity of the interview data. One teacher who participated in a telephone interview was awarded with a gift voucher of 500 NOK, while 19 teachers who participated in focus group interviews were awarded a gift voucher of 1000 NOK each.

Parents, students, and teachers, respectively, had the right to withdraw their consent at any time and have their information deleted, except for information already entered into statistical analyses.

### Measures

The primary outcomes of the study are the intervention’s acceptability and feasibility, and its effectiveness on students’ wellbeing. Background variables from the student questionnaire included gender, school grade (5–10), living in one or two homes, and parents’ country of birth (Norway or other). Family socio-economic status was assessed with the Family Affluence Scale II [17].

Our student and teacher questionnaires covered different categories of implementation outcomes, as shown in Table I. We also asked the teachers what challenges they experienced when teaching 5Ways@School, and for advice on how to improve the teaching material; the answers to these questions were in free text. An interview guide was developed for the interviews with the teachers, as summarized in Table II. In Table III, we present the wellbeing and mental health measures used to assess the effectiveness of the intervention.

**Table II.** Interview guide for qualitative interviews with teachers.

Main theme	Example questions
Tell us about your work experience as a teacher, including your perception of students’ mental health as part of the school day before the 5Ways@School programme.	• Number of years as a teacher, courses/training in mental health. • Types of mental health challenges students may face and how you address them.
What was your experience with the preparation for 5Ways@School and how did the teaching go?	• How was the training; too much/too little, missing elements? • Did you enjoy teaching the students in 5Ways@School? Why/why not?
What is your perception of any impact 5Ways@School has had on the students and yourself as a teacher?	• What has the teaching meant for the class environment and students’ wellbeing? • Could you give examples of students’ positive or negative reactions to 5Ways@School?
If 5Ways@School were to be continued at your school, or initiated at another school, what is important to continue with, and what is important to change?	• How can 5Ways@School become the best possible teaching programme for your school? • Which students is it best/least suited for?
What is your opinion about the school providing students with mental health promoting knowledge and skills, and how does this fit into the school day?	• How have you previously taught the topic ‘health and life skills’? • Who should teach this topic; teachers or other personnel?

**Table III.** Overview of measures of effectiveness.

Main category	Scales and sample items
Subjective wellbeing	Present and expected future global life satisfaction: The Cantril Ladder Scale [19]. Satisfaction with different life domains/arenas: E.g. ‘How satisfied are you with your school?’
Social relationships	A selection of items on relationships: E.g. ‘Do you have a friend whom you can trust and talk to about everything?’ ‘Do you have [a friend] to be with during 1) your leisure time and 2) in the school breaks?’
Loneliness	Single item: ‘During the past week (. . .) have you felt lonely?’
Mental health	Kandel and Davies’s six-item Depressive Mood Inventory [20]: E.g. ‘During the past week, have you felt unhappy, sad, or depressed?’ The Prosocial Subscale of the Strengths and Difficulties Questionnaire [21, 22]: E.g., ‘I often volunteer to help others (parents, teachers, children)’.
Leisure activities and screen time	Two items: ‘In your leisure time, how often do you 1) meet with friends in their homes, 2) hang out with friends [outdoor], 3) exercise or do sports’, etc. ‘In your leisure time, how may hours a day do you 1) watch tv, tv series, and/or YouTube, 2) read books, 3) play computer games, 4) play games on your smartphone, 5) use social media, 6) read/watch news?’

### Data analysis

#### Analysis of quantitative data

Quantitative data will be explored with descriptive analyses, and the effects of the intervention will then be investigated using different multivariate approaches. One such approach is multilevel regression models, in which individual measurements (level 1) at different timepoints are nested within students (level 2), which in turn are nested within school classes (level 3), in turn nested within schools (level 4). To determine if the intervention effects vary for individuals with e.g. lower wellbeing scores or higher mental health symptoms at baseline, we will stratify the sample by these scores and conduct subgroup analyses. To explore moderating effects stemming from background variables, we will add interaction terms into the multilevel models. If significant interaction terms are observed, we will conduct stratified subgroup analyses to better understand the intervention’s differential effects.

Intervention effects will also be examined using network intervention analysis [23], a novel statistical method used to understand the complex relationships between an intervention and different variables like symptoms, behaviours, or cognitive functions. In network analysis, variables are represented as nodes in a network, with the connections (edges) between them indicating relationships or interactions. The network approach is also suited to explore moderation and mediation.

Since attrition is common in longitudinal studies, we will explore the level and significance of attrition. We will investigate whether there is systematic attrition by performing a series of *t*-tests on the baseline data, followed by sensitivity analyses to examine how sensitive our results are to such attrition. We will explore different deletion and imputation methods to address missing data and evaluate their effectiveness in preserving data integrity and minimizing bias.

#### Analysis of qualitative data

Qualitative data from free text questionnaire responses will be analysed using elements from ‘small-q’ thematic analysis (TA) [24]. Small-q qualitative research reflects the use of techniques of qualitative data collection and analysis within a framework of (post)positivism. The teacher interviews will be analysed using reflexive thematic analysis [24], with focus on context and researcher reflexivity. Reflexive thematic analysis is a flexible and widely used qualitative analytic method within psychology and related fields, particularly useful for exploring complex phenomena and understanding participants’ experiences, thoughts, or behaviours [24].

## Discussion

This protocol describes the 5Ways@School effectiveness and implementation trial, carried out in Moss Municipality, Norway. The first participants consented to participate on 18 November 2023, and the last data collection took place on 13 December 2024. Data are currently being analysed.

School-based mental health promoting interventions show considerable promise [4] but often face barriers such as budget limitations, stigma, and parent consent [25]. They also commonly require substantial resources, such as external personnel, which can be costly and leave resource-strapped schools unable to support their students effectively. Curriculum-based reforms and interventions, like the 5Ways@School, are embedded within the existing educational framework, leverage current resources and infrastructure for implementation, and may constitute a more sustainable and accessible option for promoting mental health and wellbeing in schools. They have the advantage of reaching all students systematically and may help normalize dialogue surrounding mental health and wellbeing. This integration into regular classroom activities reduces stigma and equips all students with essential skills to handle stress and foster their own wellbeing.

With fewer resources and increasing needs, schools should pursue the most cost-effective strategies for improving students’ wellbeing. Structured wellbeing lessons in schools are, according to the LSE Centre for Economic Performance, among the most cost-effective wellbeing initiatives, with a ratio of benefits to net costs of 27:1 [26]. In the current project we will investigate both the costs of implementing the 5Ways@School and its benefits, so that school leaders may discuss whether the teaching material should be part of the future curriculum.

The study has the potential to generate new and valuable insights into the implementation of a school intervention within a Norwegian school setting, specifically focusing on the applicability of the Five Ways to Wellbeing framework. Its findings could also offer valuable insights relevant beyond Norway. Furthermore, the study will investigate the effectiveness of the intervention, identify plausible mechanisms of effect, and examine potential unforeseen, negative side effects. The insights gained from the implementation will be used to inform the evaluation of its effectiveness and to guide optimization of the teaching programme – both in terms of its content and its implementation – for future use in schools.

### Limitations and strengths

Our study affords both strengths and caveats, with the key limitations stemming from the necessity to tailor the research design to fit within the constraints of the intervention setting. The optimal design to investigate the effectiveness of our intervention would have been a cluster-RCT, recruiting a large number of schools in different municipalities. This was not feasible due to limited resources. As our study is a non-RCT, it is more susceptible to confounding factors and biases, and the results should be interpreted with caution. Furthermore, our attempt at standardizing the intervention, instructing the teachers to refrain from adjusting it to their students’ needs, may pose a limitation in our study, potentially negatively affecting the acceptability and effectiveness of the intervention. Another limitation concerns the use of qualitative methods being restricted to the teachers. Again, the choice to collect only quantitative data from the students was based on the financial and human resources available. A third limitation is the short time interval between the intervention start for the first and second group of students. A longer time interval would have provided more comprehensive data for comparison against the control. However, we had to conform to the academic calendar, which necessitated a shorter interval between the two phases. A final limitation is the attrition of participants over the 12-month study period, which could have been mitigated through preventive measures. For instance, we could have piloted students’ responses to repeated questionnaires to identify potential challenges and improve retention.

One major strength of our study relates to its real-life school setting and its dual focus on effectiveness and implementation. Our use of mixed methods will provide richer insights than either quantitative or qualitative methods alone. Another strength of our study is the utilization of questionnaire instruments previously employed in surveys among children and adolescents in Norway. This enables us to effectively compare our findings with national survey data. A third strength is the large number of participating students and teachers in our study, yielding a large amount of data on the implementation process and allowing us to detect even modest effects of the intervention.

In conclusion, our study aims to provide valuable insights into the effectiveness and implementation of a curricular wellbeing-promoting intervention within a school setting. Should the 5Ways@School intervention prove to be both effective and feasible, it holds potential to positively contribute to the future wellbeing of children and young people.

## Supplemental Material

sj-docx-1-sjp-10.1177_14034948251370109 – Supplemental material for Study protocol for 5Ways@School – An implementation and effectiveness trial of a school-based wellbeing intervention in 16 schools in Norway Supplemental material, sj-docx-1-sjp-10.1177_14034948251370109 for Study protocol for 5Ways@School – An implementation and effectiveness trial of a school-based wellbeing intervention in 16 schools in Norway by Kristian Green Krogshus, Espen Bjertness, Nikolai Olavi Czajkowski, Rubén Rodriguez-Cano and Ragnhild Bang Nes in Scandinavian Journal of Public Health

sj-docx-2-sjp-10.1177_14034948251370109 – Supplemental material for Study protocol for 5Ways@School – An implementation and effectiveness trial of a school-based wellbeing intervention in 16 schools in Norway Supplemental material, sj-docx-2-sjp-10.1177_14034948251370109 for Study protocol for 5Ways@School – An implementation and effectiveness trial of a school-based wellbeing intervention in 16 schools in Norway by Kristian Green Krogshus, Espen Bjertness, Nikolai Olavi Czajkowski, Rubén Rodriguez-Cano and Ragnhild Bang Nes in Scandinavian Journal of Public Health
