# Supplementary material for: Study protocol for 5Ways@School – An implementation and effectiveness trial of a school-based wellbeing intervention in 16 schools in Norway
Source: Scand J Public Health. 2025 Sep 8;54(7):692–9. doi: 10.1177/14034948251370109 (PMC13589789; doi:10.1177/14034948251370109)
Supplement: Supplementary file 1 [file sj-docx-1-sjp-10.1177_14034948251370109.docx]

Supplementary File – Five Ways @ School

Data management and availability

Data from questionnaires and interviews will be stored and analysed in TSD, which is a platform for collecting, storing, analysing, and sharing sensitive data in compliance with the Norwegian privacy regulation. TSD offers virtual servers and backup and storage systems with very high safety standards.

To complete questionnaires, students and teachers will be invited sign in through their digital Feide school account. Feide is the national solution for secure login and data sharing in education and research in Norway. With Feide, students, researchers and teachers get safe access to a number of different digital services with one username and password. TSD will ensure that every Feide username will be automatically encrypted with an encryption key unique to the project, and the encrypted username will act as the student's or teacher’s serial number in the data files. After the student data collection was initiated, we realised that to perform multilevel models, we would need information on which students were in the same school class and which school classes belonged to the same school. Since we had not collected this information with our questionnaires, Moss Municipality in collaboration with TSD provided a list of the encrypted usernames matched with de-identified school classes and de-identified school names. This additional data collection was permitted by the Regional Ethics Committee and the project’s Data Protection Officers.

The data files are located on a secure server at TSD and do not contain identifiers such as names, Feide usernames or national ID numbers. The PI and the researchers of the project will be given access to the data file to analyse data on TSD's server. The researchers will not be able to identify individual students or teachers on the basis of the data files.

Only the PI and researchers on this project have access to the datasets. The datasets generated and/or analyzed are not publicly available due to the General Data Protection Regulation (GDPR) and Norwegian research regulations. A data monitoring committee (DMC) was not found found indicated, due to the restricted amount and time period of data collection in this project, and that the study involves an intervention that pose minimal risk to participants.

## Sample size considerations

When preparing this study, we aimed to determine whether we could recruit a sufficient number of students to reliably detect the effects of the intervention. By "effect," we refer to a difference, expressed as Cohen's *d*, between the intervention group and the control group regarding students' wellbeing and mental health, attributable to the intervention. Based on published meta-analyses^1-4^, we considered Cohen's *d* = 0.16 as a conservative estimate of the intervention's effect on measures such as Kandel and Davies’s six-item Depressive Mood Inventory. We aimed for power of 80 % to detect an effect, assuming a school-level intra-cluster correlation coefficient (ICC) of 0.05, which was the median value in a systematic review of school-based cluster randomized controlled trials^5^. Studies using active consent, such as ours, typically report participation rates averaging around 67%^6^, and we therefore estimated a 70% participation rate among the 3,300 eligible students. Using a power analysis tool available at http://psychstat.org/crt2arm, and assuming 15 participating students per school class, 156 classes, an effect size of 0.16, an ICC of 0.05, and a significance level of 0.05, we achieved a power of 84%. This indicates that our sample size would be adequate for detecting an intervention effect. However, the actual participation rates in the data collections were between 39 % and 74 %, see Figure 1. Based on response from headmasters, the attrition was mainly due to the students growing tired of completing questionnaires.

Dissemination policy and publishing plan

We will follow open science and replicability practices. The project will commit to unrestricted open access to the published output of the research, and publications will be in accordance with the ICMJE recommendations and the CONSORT statement.

Planned scientific communications include 6 peer-reviewed, international publications, 3 conference participations, 1 PhD dissertation, and 2 Master theses.

Non-scientific dissemination from the beginning of the project using radio, TV, newspapers, and project’s webpage, and through social media. The trial results will be communicated via reports to participants, parents, local politicians, and to the municipality’s administration. The teaching material will be made available for other municipalities. We grant public access to the full research protocol, and to the statistical code (upon request).

Potential harms and benefits for the participants

Public health interventions, despite their best intentions, may have adverse effects^7^. For instance, our intervention's focus on social relations could inadvertently increase feelings of loneliness in some students, leading to psychological harm. Furthermore, approaches that target health behaviour, such as ours, are especially prone equity harms: the intervention might benefit most those who need it least, thereby exacerbating existing health inequalities^7^. Lastly, the time allocated to the intervention could have been better spent on other learning activities, representing an opportunity cost.

The *5Ways@School* intervention was built on existing teaching material currently used among adults at Norwegian Healthy Lifestyle Centres. Experiences from teaching the Five Ways to Well-being at Healthy Lifestyle Centres have been positive, and no harm among adult participants has been reported. To our knowledge, no harm has been reported when using the Five Ways among children and youth in other countries. Based on former studies, we expected the participants to experience benefits from the intervention, and we believed the risk of adverse reactions to the teaching programme to be very low. Likewise, we believed the risk of adverse reactions to completing questionnaires was similarly low. Nevertheless, we will investigate whether some pupils can have negative reactions, either to the teaching programme or the questionnaires. Just before the intervention period began, we gathered all the municipality's teachers, school nurses, school leaders, and other school staff (N=600) for a full day of joint training and review of the research project. During this review, we informed the school nurses about their role in supporting students if they reacted to our teaching or survey. The teachers who administered the intervention, and who knew the students in their class well, would identify if students needed further support and would refer them to school nurses if indicated. Furthermore, we investigated if there could be adverse reactions to our intervention by including a questionnaire item in the students’ questionnaire: «The teaching made me sad, afraid, or angry.» In total, we consider the cost-benefit ratio to be favourable for all participants.

Plans for communicating important protocol modifications to relevant parties

During the study, important protocol modifications were communicated by the authors RBN and KGK to relevant parties.

References

1.	Tanner-Smith EE, Durlak JA and Marx RA. Empirically Based Mean Effect Size Distributions for Universal Prevention Programs Targeting School-Aged Youth: A Review of Meta-Analyses. *Prevention Science* 2018; 19: 1091-1101. DOI: 10.1007/s11121-018-0942-1.

2.	Taylor RD, Oberle E, Durlak JA, et al. Promoting Positive Youth Development Through School-Based Social and Emotional Learning Interventions: A Meta-Analysis of Follow-Up Effects. *Child Dev* 2017; 88: 1156-1171. DOI: 10.1111/cdev.12864.

3.	Werner-Seidler A, Perry Y, Calear AL, et al. School-based depression and anxiety prevention programs for young people: A systematic review and meta-analysis. *Clin Psychol Rev* 2017; 51: 30-47. 20161024. DOI: 10.1016/j.cpr.2016.10.005.

4.	Cohen KA, Ito S, Ahuvia IL, et al. Brief School-Based Interventions Targeting Student Mental Health or Well-Being: A Systematic Review and Meta-Analysis. *Clinical Child and Family Psychology Review* 2024. DOI: 10.1007/s10567-024-00487-2.

5.	Parker K, Nunns M, Xiao Z, et al. Characteristics and practices of school-based cluster randomised controlled trials for improving health outcomes in pupils in the United Kingdom: a methodological systematic review. *BMC Medical Research Methodology* 2021; 21: 152. DOI: 10.1186/s12874-021-01348-0.

6.	Blom-Hoffman J, Leff SS, Franko DL, et al. Consent Procedures and Participation Rates in School-Based Intervention and Prevention Research: Using a Multi-Component, Partnership-Based Approach to Recruit Participants. *School Mental Health* 2009; 1: 3-15. DOI: 10.1007/s12310-008-9000-7.

7.	Lorenc T and Oliver K. Adverse effects of public health interventions: a conceptual framework. *J Epidemiol Community Health* 2014; 68: 288-290. 20131213. DOI: 10.1136/jech-2013-203118.
