# Supplementary material for: Study protocol for 5Ways@School – An implementation and effectiveness trial of a school-based wellbeing intervention in 16 schools in Norway
Source: Scand J Public Health. 2025 Sep 8;54(7):692–9. doi: 10.1177/14034948251370109 (PMC13589789; doi:10.1177/14034948251370109)
Supplement: Supplementary file 2 [file sj-docx-2-sjp-10.1177_14034948251370109.docx]

**The TIDieR (Template for Intervention Description and Replication) Checklist*:**

Information to include when describing an intervention and the location of the information

| **Item number** | **Item** | **Where located **** | |
| --- | --- | --- | --- |
|  |  | Primary paper  (page or appendix  number) | Other ^†^ (details) |
|  | **BRIEF NAME** |  |  |
| **1.** | 5 Ways @ School (Norwegian: Fem gode vaner – Hverdagsglede Skole) | ___1, 8_______ | ______________ |
|  | **WHY** |  |  |
| **2.** | 5 Ways @ School is a universal teaching programme aimed at children and adolescents in 5^th^ to 10^th^ grade (10 to 16 years). It builds on the Five Ways to Wellbeing framework and seeks to promote wellbeing and mental health through the practice of five evidence-based actions: to connect, be physically active, take notice, keep learning, and give. | ___3-4_______ | _____________ |
|  | **WHAT** |  |  |
| **3.** | The intervention consists of six weekly lessons, one for each “action” plus a review lesson. Each lesson is 90 minutes long. These lessons are available for the teachers through a web portal, and the lessons consist of introductory texts (with the option to choose between two different levels of difficulty), videos, activities, and templates for exercises that can be printed and given to the students. The teachers had access to a manual that explained the lessons in further detail, and they received a deck of discussion cards to be used in lessons. The materials can be accessed at <https://skolerom.no/velkommen-til-hverdagsglede-skole-barnetrinn/> and <https://skolerom.no/velkommen-til-hverdagsglede-skole-ungdomstrinn/>. | _____8_______ | _____________ |
| **4.** | Procedures: The intervention was developed by the publisher together with two of the researchers (KGK and RBN), personnel from the school administration, four teachers, and four students. Prior to initiation of the full scale trial in Moss Municipality, the intervention was piloted in seven school classes in Råde Municipality, and the publisher made minor adjustments based on feedback from the pilot teachers. The research project including the intervention was discussed with municipal stakeholders: the Headmasters of all 16 schools, the Youth Advisory Council, the Political Committee for Education, the Political Committee for Health Services, the organization Mental Health, and the labour organizations. | _____8_______ | _____________ |
|  | **WHO PROVIDED** |  |  |
| **5.** | The teaching was provided by 156 teachers, more specifically the *contact teachers*: In Norway, every school class has a main teacher, called contact teacher, who has the main responsibility for the practical, administrative, and socio-pedagogical tasks concerning individual students in the class. The researchers (KGK and RBN) gathered all teachers, school health nurses, and other school personnel in Moss Municipality (N=600) in a full day seminar on the research project, its aims and procedures; and the teaching programme was presented by the publisher. The contact teachers were given access to the teaching material some days prior to this seminar. | ______8______ | _____________ |
|  | **HOW** |  |  |
| **6.** | The teachers delivered the intervention in an ordinary classroom setting, face-to-face, to the whole school class. | ______7______ | _____________ |
|  | **WHERE** |  |  |
| **7.** | The intervention was given in classrooms, at 16 public schools in Moss Municipality. All classrooms had audiovisual facilities for presenting the teaching material on screen. | ______7______ | _____________ |
|  | **WHEN and HOW MUCH** |  |  |
| **8.** | The intervention was delivered as two school hours (i.e., 90 minutes) per week over six consecutive weeks, that is, a total of 12 school hours. Due to the research design, approximately 50 % of the students received the intervention first, from 20^th^ November 2023, while the remaining 50 % of the students received the intervention from January, 2024. | _____8_______ | _____________ |
|  | **TAILORING** |  |  |
| **9.** | The teachers were encouraged to adhere to the teaching manual. The intervention was not planned to be personalised, titrated or adapted; however, the teachers may have made minor adjustments to the meet the needs of their students. | _____8_______ | _____________ |
|  | **MODIFICATIONS** |  |  |
| **10.^ǂ^** | Minor adjustments to the intervention were performed after the piloting in Råde Municipality. No modifications were performed during the course of the full scale trial in Moss Municipality. | ______7______ | _____________ |
|  | **HOW WELL** |  |  |
| **11.** | Planned: Intervention adherence (fidelity) was assessed with three items in a questionnaire given to all teachers who delivered the intervention. | _____7, 9_____ | _____________ |
| **12.^ǂ^** | Actual: If intervention adherence or fidelity was assessed, describe the extent to which the intervention was delivered as planned. | N/A (protocol) | _____________ |

** **Authors** - use N/A if an item is not applicable for the intervention being described. **Reviewers** – use ‘?’ if information about the element is not reported/not sufficiently reported.

† If the information is not provided in the primary paper, give details of where this information is available. This may include locations such as a published protocol or other published papers (provide citation details) or a website (provide the URL).

ǂ If completing the TIDieR checklist for a protocol, these items are not relevant to the protocol and cannot be described until the study is complete.

* We strongly recommend using this checklist in conjunction with the TIDieR guide (see *BMJ* 2014;348:g1687) which contains an explanation and elaboration for each item.

* The focus of TIDieR is on reporting details of the intervention elements (and where relevant, comparison elements) of a study. Other elements and methodological features of studies are covered by other reporting statements and checklists and have not been duplicated as part of the TIDieR checklist. When a **randomised trial** is being reported, the TIDieR checklist should be used in conjunction with the CONSORT statement (see [www.consort-statement.org](http://www.consort-statement.org)) as an extension of **Item 5 of the CONSORT 2010 Statement.** When a **clinical trial** **protocol** is being reported, the TIDieR checklist should be used in conjunction with the SPIRIT statement as an extension of **Item 11 of the SPIRIT 2013 Statement** (see [www.spirit-statement.org](http://www.spirit-statement.org)). For alternate study designs, TIDieR can be used in conjunction with the appropriate checklist for that study design (see [www.equator-network.org](http://www.equator-network.org)).
